# Les aspects cliniques et thérapeutiques des thrombophlébites cérébrales du post-partum

**DOI:** 10.11604/pamj.2016.25.248.9576

**Published:** 2016-12-21

**Authors:** Barhoumi Mohamed Hafed, Ragmoun Houcem, Saidi Wassim, Guizani Taieb, Manssouri Waddah, Liouane Mohamed, Fatnassi Mohamed Ridha

**Affiliations:** 1 Service d’Anesthésie Réanimation, CHU Ibn El Jazzar, Kairouan, Tunisie; 2 Service de Gynécologie Obstétrique- CHU Ibn El Jazzar, Kairouan, Tunisie; 3 Clinique de Radiologie de Kairouan, Tunisie

**Keywords:** Thrombophlébites cérébrales, post-partum, IRM cérébrale, convulsion, héparinothérapie, Cerebral thrombophlebites, postpartum, brain MRI, convulsion, heparin therapy

## Abstract

Les thrombophlébites cérébrales sont des pathologies rares (1/5000 naissances) mais redoutables. La grossesse et le post-partum en sont des circonstances favorisantes: leur symptomatologie clinique est divertie et variée dominée par les céphalées, les convulsions et les déficits neurologiques mais aucun signes n’est spécifique. L’examen physique est souvent pauvre et prêt à confusion avec de nombreuses autres affections. Le diagnostic de certitude ne peut être que neuroradiologique. L’IRM cérébrale est actuellement la méthode de référence, elle permet la visualisation du thrombus veineux et le suivie de son évolution. Le traitement de ces TPC est essentiellement médical basé sur les anticoagulants. A l’occasion de quatre observations de TPC, du post-partum et d’une revue de la littérature, nous allons mettre le point sur l’importance d’un diagnostic précoce et d’une prise en charge thérapeutique adéquate.

## Abstract (en)

Cerebral thrombophlebites are rare but life-threatening conditions (1/5000 births). Pregnancy and postpartum are predisposing factors: clinical presentation vary and may be misleading, dominated by headaches, convulsions and neurological deficits but showing no specific features. Physical examination often generates informations unavailable and misleading because cerebral thrombophlebites share symptoms with other affections. A definitive diagnosis can be made only neuroradiologically. Brain MRI is currently the reference method; it allows visualization of the venous thrombus and its evolution. The treatment of cerebral thrombophlebites is essentially medical based on anticoagulant drugs. We here report four cases with post partum cerebral thrombophlebites. The aim of our study and review of the literature is to highlight the importance of early diagnosis and adequate therapy.

## Introduction

Les thrombophlébites cérébrales (TPC) sont des pathologies rares mais redoutables. Elles regroupent les thromboses des sinus veineux de la dure mère et des veines cérébrales superficielles et profondes. Sa fréquence est estimée de 1/3000 à 1/10000 naissances et 0.5 à 1% de tous les accidents vasculaires cérébrales [1]. En dehors des facteurs de risque classiques tels que l’hypercoagulabilité de la grossesse et du post-partum ainsi que la présence thrombophilie, l’impact d’autres facteurs favorisants notamment l’âge, la parité, les conditions de l’accouchement demeure imprécis. A l’occasion de quatre observations de TPC du post-partum et d’une revue de la littérature, nous allons mettre le point sur l’importance d’un diagnostic précoce et d’une prise en charge thérapeutique adéquate.

## Patient et observation

### Observation 1

**Figure 1:**
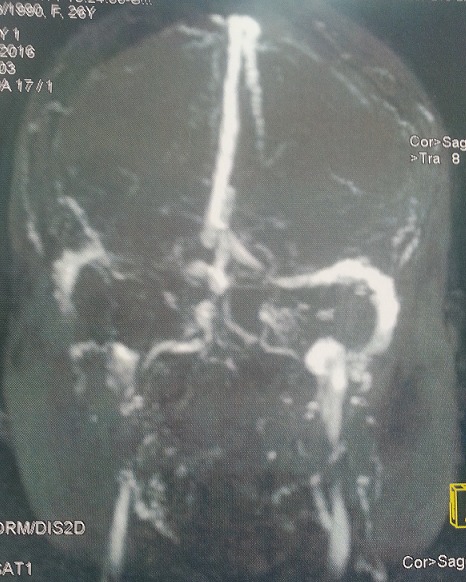
Angio IRM: thrombose au dépend du sinus transverse gauche

Patiente âgée de 22 ans, sans antécédents pathologiques notables, G1P1, était admise au 18^ème^ jour du post-partum dans un tableau d’agitation psychomotrice extrême. Le début de la symptomatologie était au 7^ème^ jour du post-partum associant des céphalées et insomnie suivie de l’installation d’un syndrome délirant et agitation psychomotrice. Elle était hospitalisée initialement au service de psychiatrie pour suspicion de psychose puerpérale et mise sous neuroleptiques mais sans amélioration. L’examen clinique initial n’objectivait pas de déficit moteur ni de raideur méningée. La tomodensitométrie cérébrale était normale ainsi que la ponction lombaire et le fond d’œil. Devant l’aggravation du tableau neurologique nécessitant une ventilation assistée. Une IRM cérébrale et l’angiographie par résonnance magnétique cérébrale avaient montré un aspect compatible avec une TPC aux dépens des sinus transverses gauches sans remaniements parenchymateux associés (Figure 1). La patiente était mise sous traitement anticoagulant à dose curative associée à un traitement anti convulsivant. L’évolution était favorable autorisant le réveil, le sevrage ventilatoire et la sortie de la réanimation au 6^ème^ jour. Un relais par les antivitamines K était entamé après 14 jours d’héparinothérapie. Vue à trois mois de traitement, la patiente ne gardait aucune séquelle fonctionnelle.

### Observation 2

Mme Z.G âgée de 43 ans, multipare, sans antécédents pathologiques notables, était admise en réanimation 32 jours après une césarienne pour SFA dans un tableau d’agitation et de confusions associées à des mouvements toniques, une perte de connaissance et morsure de la langue sans fièvre. La TDM cérébrale était normale ainsi que la ponction lombaire. Malgré le traitement anticonvulsivant, l’évolution était marquée par la persistance des crises toniques ayant nécessité sa mise sous ventilation mécanique avec une sédation profonde. Une IRM cérébrale était demandée en urgence révélant une thrombophlébite cérébrale (Figure 1). Une héparinothérapie curative avait été entamée permettant ainsi le sevrage ventilatoire et l’extubation de la patiente après 03 jours de ventilation mécanique. L’héparinothérapie était relayée après 10 jours par les antivitamines K. Elle avait quitté l’hôpital après 15 jours sans séquelles.

### Observation 3

Patiente âgée de 29 ans, G3P2, sans antécédents pathologiques particuliers, avait consulté pour céphalées, vomissement et trouble de la conscience. L’examen avait révélé un score de Glasgow de 11/15, une désorientation temporo-spatiale, ROT vifs, des chiffres tensionnels élevés 18/9 et un OMI de type rénal. Devant ce tableau de pré éclampsie sévère, la patiente a reçu une dose de charge de 4g de sulfate de magnésium en IVL entretenu par 1g/h à la pousse seringue électrique associée à 1 mg/h de Loxen. L’échographie pelvienne avait montré un Hématome rétroplacentaire compliqué d’une MFIU. Le bilan biologique n’avait pas montré d’anomalies particulières. La patiente avait subi une césarienne pour sauvetage maternel. Elle était mise sous sulfate de Magnésium à la dose de 1g /h et du Loxen à la pousse seringue électrique. L’évolution était marquée par l’apparition d’une crise convulsive tonico-clonique survenue deux heures du post opératoire et cédée par 10 mg de Valium. Le bilan post opératoire immédiat avait révélé une CIVD avec une Hb 8g/dl, Ht à 21%, une thrombopénie à 47000, TP à 45% et une fibrinémie < 0.8g/l. Elle avait bénéficié d’une transfusion de deux culots globulaires, 6 CP, 6 PFC et 4g de fibrinogène. Le bilan de contrôle avait montré: Hb à 7.5 g/dl, Ht à 23%, plaquette à 63.000, TP à 95% et Fib à 3.05g/l. Une amélioration clinique et biologique était notée. A J2 post opératoire, la patiente avait présenté une altération de l’état de conscience, un Glasgow à 13/15 avec une dysarthrie. Un scanner cérébral d’urgence avait révélé une hypodensité spontanée de deux capsules externes évoquant une thrombophlébite cérébrale qui était confirmée par une IRM cérébrale. La patiente était mise sous héparinothérapie à dose curative. L’évolution était marquée par l’amélioration de l’état neurologique. Un chevauchement avec AVK était entamé au 5^ème^ jour. La patiente avait quitté le service sans séquelles avec une INR à 2.26 et une TP à 38%.

### Observation 4

**Figure 2:**
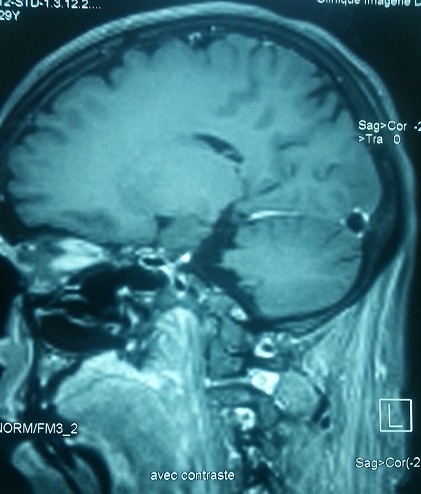
TDM cérébrale: foyer de ramollissement veineux pariétal gauche siège d’une hémorragie intra lésionnelle

**Figure 3:**
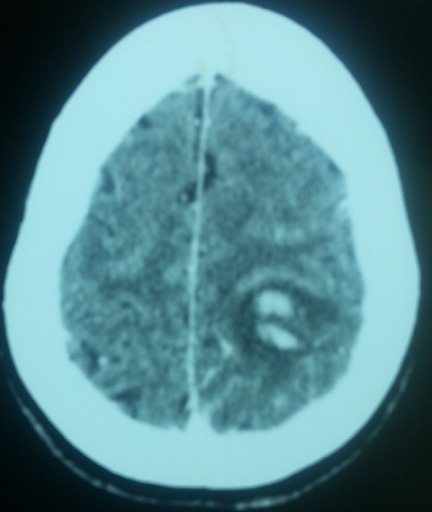
Une IRM cérébrale révélant une thrombophlébite cérébrale au dépend du sinus longitudinale supérieur avec un ramollissement hémorragique d’un infarctus veineux pariétale postérieure gauche

Patiente âgée de 26 ans, sans antécédents pathologiques notables, G1 P1 était admise en réanimation à 17 jours du post-partum dans un tableau d’agitation, trouble du comportement et fièvre, suivi d’une altération de l’état de conscience. L’examen clinique initial montrait une anisocorie, score de Glasgow à 10/15, une hémiplégie droite et une paralysie faciale gauche. L’évolution était marquée par l’aggravation rapide de l’état de conscience nécessitant son intubation et la ventilation mécanique. L’exploration par une TDM cérébrale a mis en évidence un foyer de ramollissement veineux pariétal gauche qui est le siège d’une hémorragie intra lésionnelle (Figure 2). Une IRM cérébrale était demandée en urgence révélant une thrombophlébite cérébrale au dépend du sinus longitudinale supérieur avec un ramollissement hémorragique d’un infarctus veineux pariétal postérieur gauche (Figure 3). Elle avait bénéficié d’une sédation profonde avec une optimisation de son état hémodynamique et mise sous une anticoagulation curative. Elle avait bénéficié également d’un volet crânien gauche décompressif qui était conservé sous le tissu adipeux de l’abdomen. L’évolution était marquée par une amélioration neurologique et respiratoire aux dépens d’un séjour en réanimation nécessitant des soins lourds et prolongés. Mise sortante après 64 jours de réanimation, consciente, score de Glasgow à 15/15 sous AVK. 6 mois plutard, elle avait bénéficié d’un repositionnement de son volet crânien.

## Discussion

Les femmes enceintes ont un risque accru de développer des thromboses veineuses cérébrales, en raison de leur état d’hypercoagulabilité, de l’augmentation des facteurs de coagulation (VII, VIII, IX, XI, XII) du fibrinogène et des plaquettes et de la diminution de l’activité fibrinolytique. Le post-partum est également une circonstance favorisante de survenue de cette complication. En effet, dans plus de la moitié des cas, ces TPC surviennent dans la première semaine des suites de couche [2]. Ce risque augmente également avec l’âge maternel, l’accouchement par césarienne ainsi que la présence d’une HTA, d’infection et de vomissements gravidiques [2, 3]. La symptomatologie clinique de ces TPC est très polymorphe. L’essentiel est d’y penser pour faire un diagnostic précoce. Leur installation peut être précoce (moins de 48h de l’accouchement) dans 28% des cas, sub aigu (entre 48h et 30 jours) dans 47% des cas ou tardive (plus de 30 jours) dans 25% des cas [2, 4]. Une TPC doit être suspectée lorsque la parturiente développe des symptômes associant à des degrés divers une hypertension intra crânienne (céphalées, vomissements, œdème papillaire, troubles de la conscience) et/ou un déficit neurologique focal et/ou des crises convulsives [3]. Les céphalées constituent le symptôme le plus fréquent, retrouvées dans 75% des cas, elles n’ont pas de caractéristiques ou de profil évolutif spécifique. Un œdème papillaire est présent environ dans 50% des cas, un déficit neurologique dans 34 à 70% des cas, des crises convulsives dans 20 à 57% des cas, des troubles de la conscience dans 34 à 70% des cas ou des troubles psychiatriques qui sont parfois au premier plan et marquent le reste de la symptomatologie [5, 6]. Nos quatre malades avaient une installation sub aiguë. Leur symptomatologie associe des signes d’hypertension crânienne et d’agitation dans 03 cas, un déficit neurologique dans un cas et crises convulsives dans 02 cas.

Cette symptomatologie clinique pose un problème de diagnostic différentiel avec une éclampsie, une hémorragie sus arachnoïdienne, un accident ischémique transitoire [7, 8] voire une psychose puerpérale [4]. comme c’est le cas de notre première observation. Le diagnostic positif ne peut être que neuroradiologique [1]. La TDM cérébrale sans et avec injection de contraste est encore réalisée en première intention. Elle reste normale chez 4 à 25% des patientes ayant une TPC [3]. L’aspect type est la présence de signe de delta, retrouvé dans environ 25% des cas. Il apparaît comme une aire hypodense entourée d’une prise de contraste. Un autre signe direct est le thrombus frais qui apparaît sous forme d’une hyperdensité spontanée à l’endroit de la veine thrombosée [9]. Les signes indirects visibles sur le scanner cérébral sont essentiellement les infarctus veineux mais aussi l’existence d’un œdème cérébral. Dans notre travail, la TDM cérébrale était normale dans les deux premières observations, montrait un infarctus veineux dans le 3^ème^ et la présence d’une thrombose veineuse dans le 4^ème^cas. L’IRM cérébrale est actuellement la méthode de référence pour le diagnostic de TPC. Les séquences habituelles sont les séquences écho et spin pondérés en T1 et T2, les séquences FLAIR pour l’étude du parenchyme. La séquence T2* sensible à la présence du sang et plus récemment les séquences pondérées en diffusion et perfusion. L’IRM permet la visualisation du thrombus veineux et le suivi de son évolution. L’IRM était pratiquée chez trois de nos patientes, avait permis le diagnostic de TPC alors que la TDM cérébrale était normale. L’angiographie par résonance magnétique [10] est un complément de l’IRM cérébrale. Elle permet la visualisation de la circulation veineuse et de la thrombose.

La recherche d’une thrombophilie est systématique pour détecter un éventuel déficit en anti thrombine III, responsable de 20 à 40% de thrombose pendant la grossesse [6] ou d’un déficit en protéine C ou S responsable respectivement de 7 à 22% de thrombose dans le post-partum [5, 7]. Le traitement des TPC comporte deux volets: le traitement du processus thrombotique se base sur l’anticoagulation dont l’objectif est de prévenir l’extension de la thrombose afin de permettre le développement d’une circulation collatérale et la prévention des infarctus veineux. Le risque théorique est celui d’une hémorragie massive au sein d’un infarctus, volontiers spontanément hémorragique. Deux essais thérapeutiques randomisés ont évalué le rapport bénéfice/risque du traitement anticoagulant contre placebo chez des patientes ayant une TPC prouvée, ont montré un bénéfice statistiquement significatif en faveur de l’héparine et sans risque majeur d’hémorragie [3, 11]. Le traitement par les fibrinolytiques est difficile à recommander en absence d’études randomisées qui permettent de comparer le rapport risque /bénéfice [1]. Le traitement symptomatique visant à lutter contre l’hypertension intra crânienne en se basant sur les traitements médicamenteux tel que les corticoïdes, le mannitol, la sédation profonde et parfois un traitement chirurgical notamment la craniotomie décompressive qui peut se discuter en cas d’œdème majeur. Le traitement anti épileptique est systématique en cas de crises épileptiques. Les facteurs classiques de mauvais pronostic sont l’atteinte des veines profondes, l’existence d’un coma et l’âge avancé [2]. L’évolution se fait vers la guérison dans la majorité des cas, les séquelles permanentes sont observées dans 10 à 30% des cas [1]. En ce qui concerne nos patients, l’évolution était bonne en raison de l’instauration rapide de l’héparinothérapie et d’une prise en charge adéquate de l’hypertension intra crânienne.

## Conclusion

Les TPC en post-partum est une pathologie rare mais grave. Le diagnostic clinique reste difficile, il faut savoir y penser même devant des signes peu évocateurs et demander des examens neuro radiologiques pour le confirmer notamment l’IRM. Le bénéfice de l’héparinothérapie est maintenant bien demandé. Le pronostic reste bon si le diagnostic est fait à temps et si le traitement est entamé précocement.
